# Translational Control of *Xenopus* Oocyte Meiosis: Toward the Genomic Era

**DOI:** 10.3390/cells9061502

**Published:** 2020-06-19

**Authors:** Ferdinand Meneau, Aude Dupré, Catherine Jessus, Enrico Maria Daldello

**Affiliations:** Sorbonne Université, CNRS, Laboratoire de Biologie du Développement—Institut de Biologie Paris Seine, LBD—IBPS, F-75005 Paris, France; ferdinand.meneau@etu.upmc.fr (F.M.); aude-isabelle.dupre@upmc.fr (A.D.); catherine.jessus@upmc.fr (C.J.)

**Keywords:** *Xenopus* oocytes, meiotic maturation, translation, mRNA polyadenylation

## Abstract

The study of oocytes has made enormous contributions to the understanding of the G_2_/M transition. The complementarity of investigations carried out on various model organisms has led to the identification of the M-phase promoting factor (MPF) and to unravel the basis of cell cycle regulation. Thanks to the power of biochemical approaches offered by frog oocytes, this model has allowed to identify the core signaling components involved in the regulation of M-phase. A central emerging layer of regulation of cell division regards protein translation. Oocytes are a unique model to tackle this question as they accumulate large quantities of dormant mRNAs to be used during meiosis resumption and progression, as well as the cell divisions during early embryogenesis. Since these events occur in the absence of transcription, they require cascades of successive unmasking, translation, and discarding of these mRNAs, implying a fine regulation of the timing of specific translation. In the last years, the *Xenopus* genome has been sequenced and annotated, enabling the development of omics techniques in this model and starting its transition into the genomic era. This review has critically described how the different phases of meiosis are orchestrated by changes in gene expression. The physiological states of the oocyte have been described together with the molecular mechanisms that control the critical transitions during meiosis progression, highlighting the connection between translation control and meiosis dynamics.

## 1. Introduction

Meiosis is a specialized cell division that is essential for sexual reproduction in eukaryotes as it allows germ cells to reduce by half their ploidy. This process relies on the synthesis of mRNAs whose timely translation and/or degradation drives the differentiation of the oocyte into a fertilizable egg. These stockpiled mRNAs and proteins regulate processes beyond meiosis, such as the binding of a single sperm to the oocyte or the fusion of the maternal and paternal genomes after fertilization. Additionally, some of them are inherited by the embryo as maternal mRNA. The proteins derived from these mRNAs are used in the embryo as a source of energy, but also to define the basic embryonic axis formation, while others are critical cell cycle regulators of the first embryonic cell cycle, as well as the transcription machinery necessary for the expression of the embryonic genome.

**Figure 1:**
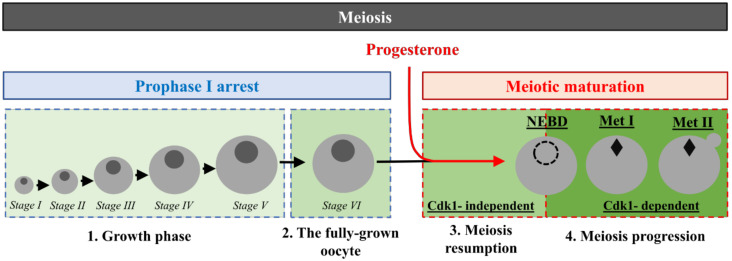
Four phases of *Xenopus* oocyte meiosis. (**1**) The growth period: Oocytes enter prophase of the first meiotic division and arrest at the diplotene stage (Prophase I). Growing oocytes have been classified into six stages by Dumont [31]. (**2**) The fully-grown oocyte: when oocyte growth is complete, transcription and vitellogenesis stop, and oocytes become competent to undergo meiotic maturation. (**3**) Meiosis resumption: Progesterone releases the prophase arrest and oocytes resume meiosis. (**4**) Meiosis progression: Following nuclear envelope breakdown (NEBD), oocytes assemble the metaphase I (Met I) spindle. After anaphase I, the first polar body is extruded, and a metaphase II (Met II) spindle assembles.

During oogenesis, female germ stem cells, or oogonia, proliferate by mitosis. After a long premeiotic S-phase, oogonia become oocytes and enter in prophase of the 1st meiotic division (prophase I). This long-lasting arrest (months to years depending on vertebrate species) is conserved in all the animal kingdom (Figure 1). It allows oocytes to accumulate RNAs, proteins, organelles, and nutrients required for meiosis and embryonic development. At the end of this growth phase, and in response to hormonal stimulation, oocytes undergo the last step of oogenesis, meiotic maturation, which consists of two consecutive divisions in the absence of DNA replication. In the vertebrates, oocytes arrest again at metaphase of the 2nd meiotic division, awaiting fertilization (Figure 1).

As in mitosis, meiotic maturation is regulated by the post-translational modifications of proteins, like phosphorylation and the translation of mRNAs. Reversible phosphorylation results from kinases and phosphatases activities, whose regulation is governed by the universal inducer of cell division, the M-phase promoting factor (MPF). MPF consists of a complex between Cyclin-dependent-kinase 1 (Cdk1) and its regulative subunit—the Cyclin B [1,2]. In prophase, MPF is indirectly kept inactive because of the high concentration of cAMP and the activity of protein kinase A (PKA) in vertebrates [3,4]. The hormonal stimulation induces a drop of cAMP level and PKA inactivation [3,4], which, in turn, signals the oocyte to resume meiosis. Meiosis resumption is thus equivalent to the G_2_/M transition of the mitotic cell cycle. In this model system, MPF activation obeys a two-step mechanism [5]. First, a small amount of active MPF is formed. While the biological events leading to the initial activation of MPF are not completely clear, de novo protein translation is required in most vertebrates, with the exception of small rodents [6]. This starting amount of active MPF initiates positive and negative feedbacks, named the MPF auto-amplification, ending with full MPF activation [5]. Fully active Cdk1 then promotes structural events of cell division, starting with the nuclear envelope breakdown (NEBD). Following chromosomes aligning on the metaphase I plate, oocytes transition to anaphase I and extrudes the first polar body. They then assemble quickly a second metaphase spindle and arrests in metaphase II. The second regulatory mechanism of meiotic maturation is the control of gene expression, which mostly relies on translation and mRNA turnover, as transcription is silent, with the exception of some foci identified in mouse [7]. Importantly, changes in gene expression occur and regulate meiosis resumption and progression by targeting mRNAs that have accumulated during early oogenesis. They shape the proteome of the oocyte by modulating mRNA stability (transcriptome), the degree of mRNA translation (translatome), and protein stability. Previous studies have identified two main changes in the gene expression program of the oocyte: the “early” wave of translation occurring downstream of the hormonal stimulation and a “late” wave occurring after NEBD. Among proteins synthesized during meiosis, three classes can be defined: Class I corresponds to a sub-group of the early translated proteins involved in MPF activation, Class II is involved in meiosis progression, and Class III is required for the embryo development (Table 1).

The oocyte is a powerful experimental system to study how translation initiation and termination are regulated during cell division. Historically, the *Xenopus* oocytes have been an excellent model to biochemically characterize the cellular mechanisms governing meiosis progression. Recently, omics datasets became available. This review has described the current knowledge of the molecular mechanisms responsible for the genome-wide changes in gene expression occurring during vertebrate meiosis, relying on the *Xenopus* model system. The regulation of gene expression allows the achievement of four major sequential steps that orchestrate oogenesis: the oocyte growth phase, the arrest of the fully-grown oocyte, meiosis resumption, from the hormonal stimulation to NEBD, and finally meiosis progression from NEBD to the metaphase II arrest.

## 2. The Early Oogenesis, a Growth Period That Sets the Stage for Meiosis Resumption

During the early phases of oogenesis, transcription is highly active. *Xenopus* oocytes undergo extensive growth (from 50 µm to 1.2 mm) and accumulate RNAs (mRNAs, rRNAs, tRNAs), organelles, nutrients, and proteins. Some of them are synthesized by the oocyte, while others are up-taken from the bloodstream [32]. Once pre-mRNAs are transcribed, the cleavage and polyadenylation specific factor (CPSF) recognizes the polyadenylation signal (PAS: AAU_1-2_AAA) in 3′UTR (3′untranslated region), cleaves the pre-mRNA, and adds a long poly(A) tail [33]. After capping and splicing, mRNAs are exported to the cytoplasm where they are either translated or stockpiled for later translation during either meiotic maturation or the embryo development. The stable but non-translated mRNAs undergo de-adenylation [32,34]. The molecular composition of the complexes inhibiting their translation is still debated (reviewed in [35,36]).

**Figure 2:**
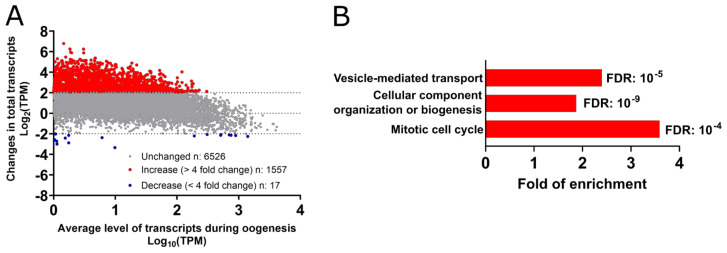
Changes in the total mRNA level during the growth period. (**A**) Changes in mRNA levels between stages I–II (starting of the growth period) and stages V–VI (end of the growth period) have been plotted. (**B**) Selection of the gene ontology terms enriched among the 1557 transcripts whose levels increase during the growth period.

Analysis of the published atlas reveals that extensive changes take place in the transcriptome during *Xenopus* oogenesis [37]. The level of 1557 transcripts increases more than 4-fold in oocytes, while the level of only 17 transcripts moderately decreases during this temporal window (Figure 2A). Gene ontology analysis reveals upregulation of transcription of genes involved in endocytosis (Figure 2B). Among these transcripts is the mRNA coding the vitellogenin receptor (very low-density lipoprotein receptor, VLDLR), whose expression increases around five times. In oocyte, VLDLR plays an essential role in the uptake of vitellogenin that is synthesized by the liver, released in the blood circulation, incorporated into the oocyte by endocytosis, and cleaved in the oocyte into yolk proteins, providing the nutrients for early embryos [38]. The oocyte extensive phase of growth is also accompanied by increased transcription of mRNAs involved in cell biogenesis and M-phase machinery (Figure 2B). Hence, the functions associated with these increased transcripts are correlated either with the growth of the oocyte or with its future activities—the meiotic and embryonic divisions. Importantly, although the meiotic cycle is arrested at diplotene of prophase I in all stages of oocyte growth, only large stage V and fully-grown stage VI oocytes are responsive to progesterone, which initiates the maturation process when physiological and environmental conditions are favorable [39] (Figure 1). The unresponsiveness of small oocytes prevents premature meiotic maturation, hence avoiding the production of haploid and fertilizable oocytes of insufficient size. In stage IV oocytes, the MPF auto-amplification loop is not functional because polo-like kinase 1 (Plk1) is not expressed at the protein level despite the presence of its mRNA [40]. Indeed, Plk1 protein accumulates between stages V and VI, when oocytes become competent to resume meiosis. These results suggest that translation during early oogenesis is temporally regulated, and the machinery required for meiosis progression is acquired progressively during oogenesis.

Obtaining the translatome of growing oocytes will help to globally sort mRNAs that are transcribed and translated immediately from the ones that are transcribed and stored for future usage. Furthermore, this will help to explore the molecular mechanisms behind the oocyte genome silencing, the spatial polarization of oocytes, and the acquisition of the competence to resume meiosis.

## 3. The Prophase-Arrest of Fully-Grown Oocytes Competent to Resume Meiosis

### 3.1. The Interplay between PKA and Protein Synthesis

It is well established that the prophase arrest of fully-grown vertebrate oocytes is maintained by high levels of cAMP and activity of the cAMP-dependent kinase (PKA) [41,42,43]. In *Xenopus* oocytes, the constitutively active Gαs-protein coupled receptor 185 (GPR185) results in high adenylate cyclase activity that maintains the high levels of cAMP [44]. Upon hormonal stimulation, the cAMP concentration drops, inducing PKA inactivation in less than one hour [42,45]. However, the ability of progesterone or the PKA-inhibitor (PKI) to induce meiosis resumption is abolished if the translation is inhibited by protein synthesis inhibitors, such as cycloheximide [45,46], suggesting that, during the prophase arrest, PKA prevents the synthesis of crucial proteins required for meiosis resumption.

### 3.2. The Substrates of PKA Mediating the Prophase Arrest

Little is known regarding the identity of the PKA substrates that lock oocytes in prophase. In *Xenopus* oocytes, the first ones reported corresponding to two proteins called MpP-20 (Maturation phospho-protein 20 kDa) and MpP-32 (32 kDa) [47]. The identity of MpP-20 was uncovered as Arpp19 (cAMP-regulated phosphoprotein), a protein that belongs to the Endosulfine family [48]. In prophase-arrested oocytes, PKA phosphorylates Arpp19 at S109 within a PKA consensus site well-conserved among eukaryotes. Under this phosphorylated state, Arpp19 blocks meiosis resumption [49]. Arpp19 phosphorylation at S109 results from an equilibrium between PKA and a recently identified phosphatase, PP2A-B55δ [50]. In prophase-arrested fully-grown oocytes, the action of PP2A-B55δ on Arpp19 is overwhelmed by PKA activity, resulting in Arpp19 phosphorylation at S109. In response to progesterone or PKI injection, PKA activity decreases, allowing PP2A-B55δ to efficiently dephosphorylate Arpp19 [49,50,51]. This event is critical for meiosis resumption as the overexpression of a phosphomimic mutant form of Arpp19 (S109D) impairs Cdk1 activation induced by either progesterone or PKI [49]. This initial function of Arpp19 as a PKA substrate is fully distinct from the second one operating at the time of MPF activation when Arpp19 becomes phosphorylated at a distinct site (S67) by the kinase Greatwall [52,53,54]. The mechanism by which PKA-phosphorylated Arpp19 inhibits Cdk1 activation has not been yet elucidated. An attractive hypothesis is that Arpp19 regulates the synthesis of proteins required for MPF activation, which relies on PKA downregulation. Interestingly, the yeast homolog of Arpp19 has been involved in RNA metabolism [55]. The identity of MpP-32 has not been solved. However, it has been proposed that MpP-32 corresponds to DARPP32 (Dopamine And CAMP-Regulated Neuronal Phosphoprotein 32), another member of the cAMP-regulated phosphoprotein family [47]. When phosphorylated by PKA, DARRP32 is a strong inhibitor of the S/T protein phosphatase-1 (PP1) [56,57,58,59]. Since PP1 activity is essential for meiosis resumption [60,61], the inhibition of PP1 by PKA-phosphorylated DARPP32 could contribute to the prophase arrest. Beside DARPP32, other specific inhibitors of PP1 are also regulated by PKA, such as inhibitor-1 that is able to prevent meiosis resumption [45]. Hence, a more in-depth investigation of the interplay between PKA and PP1 could give some hints regarding how oocytes are arrested in prophase. Two direct regulators of Cdk1—the phosphatase Cdc25 and its opposing kinase Wee1B—have been proposed as PKA substrates in oocytes [62,63,64]. In prophase-arrested oocytes, Cdc25 is phosphorylated at S287 by PKA, hence promoting its sequestration in the cytoplasm through the recruitment of 14-3-3 protein [65]. During meiosis resumption, Cdc25 is dephosphorylated at S287, but this event depends on protein synthesis and is only observed at the time of Cdk1 activation, i.e., several hours after PKA downregulation [63]. Therefore, Cdc25 unlikely corresponds to the missing link between PKA and protein synthesis. In mouse oocytes, Wee1B is phosphorylated by PKA at S15 and contributes to Cdk1 inhibition during the prophase arrest [62,64]. Since the *Xenopus* ortholog of Wee1B is not expressed in prophase oocytes, Wee1B cannot account for the arrest in prophase in this species [66,67,68,69].

The identification of the PKA substrates, which inhibit protein synthesis either directly or indirectly, is a pre-requisite to understand the mechanisms responsible for the prophase arrest. Strikingly, these proteins must meet several criteria (Table 2): (i) to be expressed in prophase; (ii) to be dephosphorylated following progesterone stimulation within one-hour, prior protein synthesis; (iii) to block progesterone or PKI-induced maturation when phosphorylated by PKA, similarly to cycloheximide. These proteins must be unable to inhibit the MPF auto-amplification process that is directly induced by either PP2A phosphatase inhibitors, such as okadaic acid, or a cytoplasmic transfer from metaphase II oocytes [1]. The analysis of the published proteome [70] reveals that nine proteins contain a peptide with a strong predicted site for PKA and whose phosphorylation decreases, 90 min after progesterone addition, by at least 50% (Table 3). Interestingly, three of these putative PKA substrates have a known function related to the control of protein synthesis—Rps6, Spats2, and Akt1s1—which are, respectively, a component of the ribosome, an RNA-binding protein involved in male meiosis, and a regulatory subunit of the master regulator of protein synthesis, mTORC1 (mTOR complex 1) [71]. These and other newly identified PKA substrates should be evaluated following the proposed criteria to get new insight into the molecular mechanism by which PKA activity selectively represses the synthesis of proteins required for Cdk1 activation.

###  3.3. Genome-Wide Description of the Prophase Arrest of Fully-Grown Oocytes

**Figure 3:**
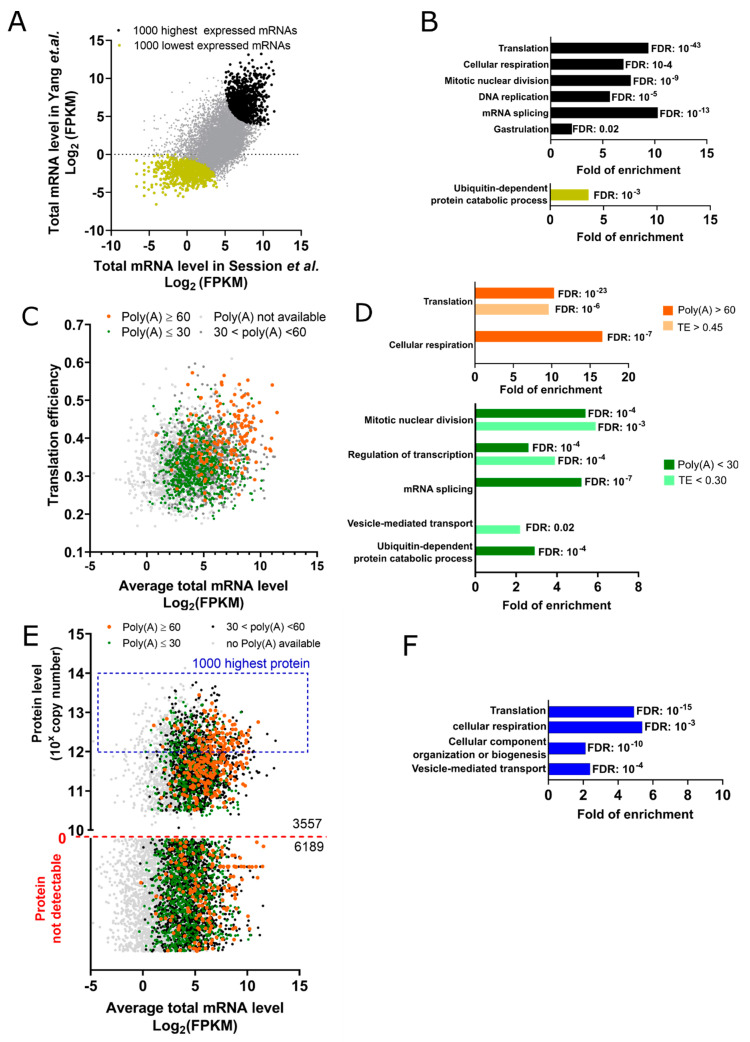
Genome-wide analysis of the fully-grown prophase-arrested oocytes. (**A**) The expression level of each mRNA quantified using two independent published datasets [37,72]. Each transcript is ranked in the two databases according to its level of expression. The average rank is used to highlight the 1000 transcripts with either the lowest (yellow) or the highest level of expression (black). (**B**) Gene ontology terms enriched among 1000 highest (black) or 1000 lowest (yellow) expressed transcripts from panel A. (**C**) The translation efficiency (TE) is defined as the fraction of mRNAs recovered in the polysomes divided by the concentration of total mRNAs of each transcript. TE has been correlated with its total level [37,72]. The extent of the poly(A) tail length has been color-coded: black (30 to 60 nt), green (≤30 nt), orange (≥60 nt), grey (poly(A) length not available). (**D**) Gene ontology terms enriched among the transcripts with poly(A) tail ≥ 60 nucleotides (dark orange) and TE ≥ 0.45 (light orange) or among poly(A) tail ≤ 30 nucleotides (dark green) and TE ≤ 30 (light green) from panel C. (**E**) The expression level of each protein is compared to the level of its mRNA [37,70,72]. The same color code as panel C is applied for the poly(A) length. (**F**) Gene ontology terms enriched among 1000 highest expressed proteins from panel E.

In the fully-grown oocyte, the nucleus is localized in the animal half of the cell and contains chromosomes that are partially condensed but transcriptionally silent. Five distinct datasets, including two transcriptomes [37,72], one analysis of mRNA poly(A) tail length [72], one translatome [72], and one proteome [70], have been published and can be used to characterize the physiological state of the fully-grown oocytes in a genome-wide manner. Since transcription is strongly downregulated, the transcriptome directly reflects cytoplasmic stockpiled mRNAs that have accumulated during early oogenesis. Because the results from the two published transcriptomes correlate well, they allow determining high and low abundant transcripts in the fully-grown oocyte (Figure 3A). Gene ontology analysis of these transcripts reveals that the lowest expressed mRNAs are enriched in components of the ubiquitin-degradation pathway, while the highest expressed transcripts are connected to translation (ribosomal proteins and translation initiation factors), energy production, as well as mitotic M- and S-phases regulation, mRNA splicing, and gastrulation (Figure 3B). Interestingly, the low level of mRNAs associated with the ubiquitin-degradation pathway suggests that protein stability is enhanced in fully-grown oocytes, as it has also been described for mRNAs [73]. In mouse oocytes, protein stability is an important mechanism to maintain oocytes arrested in prophase [74]. Hence, further studies will be required to better understand the role and regulation of protein turnover during early oogenesis and the prophase arrest in the vertebrate species.

Cytoplasmic polyadenylation is one of the main mechanisms, controlling translation during both meiosis and the first embryonic cell divisions [75]. The genome-wide correlation between translation and poly(A) tail length is, however, lost during later phases of embryo development, suggesting that additional mechanisms become functional to control translation [75]. Therefore, published datasets of the poly(A) tail length measured before this embryonic switch can be used to identify mRNAs that are actively translated from the ones that are stored for future use [72]. Indeed, mRNAs with a poly(A) tail longer than 60 nt are enriched among transcripts with a higher translation efficiency (TE) (Figure 3C). Gene ontology indicates that the highly polyadenylated transcripts in prophase-arrested oocytes, i.e., most likely actively translated, are implicated in ribosome synthesis and cellular respiration (Figure 3C,D). Conversely, mRNAs involved in mitotic nuclear division, DNA replication, and RNA splicing bear short poly(A) tails (Figure 3D), suggesting that these mRNAs categories are stored for later translation. Indeed, DNA replication and transcription are not taking place during meiotic divisions but only after fertilization, in the embryo. This translational program is well-conserved in mouse prophase-arrested oocytes [76]. However, not all the genes regulating M-phase progression are translationally repressed. mRNAs encoding the kinase Cdk1 and its regulatory subunit—Cyclin B2—are highly expressed in the prophase-arrested oocyte. Their active translation allows the formation of inactive Cdk1-Cyclin B complexes in both *Xenopus* and mouse [77,78].

The published proteome reveals that only 40% of mRNAs expressed in fully-grown oocytes are translated at a level detectable by mass spectrometry [70]. On average, mRNAs encoding proteins that are detectable by mass spectrometry are more abundant as compared to those encoding undetectable proteins (Median_(detected)_ = 20 TPM vs. Median_(not detected)_ = 10 TPM, TPM: transcript per million) (Figure 3E). Additionally, 60% of mRNAs with poly(A) tail longer than 60 nt encode proteins that are present in the proteome dataset (Figure 3E). This implies that the proteome of prophase oocytes results from both the abundance and the poly(A) tail length of mRNAs. However, 126 transcripts, which are well-expressed in fully-grown oocytes (TPM ≥ 20) and bear a poly(A) tail longer than 60 nt, are not detectable at the protein level. Conversely, 442 transcripts have short poly(A) tails but are detectable by proteomic approaches. These discrepancies between protein level and mRNAs’ polyadenylation can be explained by the regulation of protein turnover and/or by still uncovered translational regulatory mechanisms that would be independent of the poly(A) tail length. Additionally, mass spectrometry data must be interpreted with caution since some abundant proteins might not be detectable because, for example, not efficiently digested by Trypsin. More generally, detection by mass spectrometry does not allow a strict correlation of absolute copy numbers, while it still gives very precise numbers of the relative abundances. The 1000 proteins with the highest level of expression are involved in translation, cellular respiration, biogenesis of cellular components, and vesicle-mediated transport (Figure 3F). Hence, the proteome provides the identity of proteins that are highly translated in the fully-grown oocyte but also proteins that have been synthesized earlier during the growth period of oogenesis. Consistently, the vitellogenin uptake from the bloodstream is not anymore active in fully-grown oocytes [31], and the class of mRNA regulating this process bears short poly(A) tail at this final growth stage [72] (Figure 3C,D).

## 4. The Release of the Prophase Block: The Unexplored Path That Connects PKA Downregulation to MPF Activation

In most vertebrates, protein synthesis is required to activate MPF and to induce M-phase entry. In *Xenopus*, progesterone promotes the inactivation of PKA that activates de novo mRNA translation and ends with MPF activation within 3 to 5 h. How PKA downregulation leads to mRNA translation remains largely unknown.

### 4.1. De Novo Protein Translation: A Necessary Step for Meiosis Resumption

Class I proteins are present either at an extremely low level or not expressed in prophase-arrested oocytes. Their synthesis from cytoplasmic stockpiled mRNAs is required for MPF activation (Figure 1). Among Class I, three proteins have been identified so far—Cyclin B [5,16], Mos [10,11,12,13,14,15], and Ringo/Speedy [8,9]. Mos is a kinase-specific of the germline that is translated upon hormonal stimulation and indirectly activates MAPK (mitogen-activated protein kinase). While the Mos/MAPK pathway contributes to MPF activation in some vertebrates, including the amphibian *Xenopus* [10,11,12,13,14,15], this pathway is essential in all species for the formation of meiotic spindles, the inhibition of DNA replication between the two divisions, and the secondary meiotic arrest [13,14,15,79,80,81]. Ringo/Speedy activates Cdk1 in a non-canonical manner by direct binding to free Cdk1 [8,9]. The injection of either Mos, Cyclin B, or Ringo/Speedy induces meiosis resumption independently of the hormonal stimulation. However, inhibiting the synthesis of any of these proteins delays, but does not prevent, meiosis resumption upon progesterone stimulation [5,15,77]. Hence, the synthesis of any of these components is sufficient but not necessary for meiosis resumption, demonstrating the robustness of the signaling pathway induced by progesterone. Initially, it was proposed that an “early translation” of Mos, which activates MAPK, is required for meiosis resumption and necessary for the Cdk1-dependent “late translation” of Cyclin B1 [82]. However, this temporal classification contradicts other published results. First, while inhibiting protein synthesis blocks meiosis resumption in response to progesterone, preventing Mos translation or MAPK activation only delays Cdk1 activation [6,14,15,83]. This demonstrates that Mos is not the only protein whose translation is required for meiosis resumption. Second, the inhibition of Cyclin B translation with antisense strongly delays meiosis resumption in response to progesterone, hence suggesting that Cyclin B translation contributes to Cdk1 activation and does not take place downstream Cdk1 activation [5]. Additionally, in progesterone-stimulated oocytes in which Cdk1 activity is inhibited with a CDK inhibitor, p21^Cip1^, Cyclin B1 accumulates but not Mos [16]. Altogether, these results strongly argue for Cyclin B1 being an early translated mRNA. Moreover, even if Mos mRNA belongs to the early-translated mRNAs, the accumulation of Mos protein depends on Cdk1 activation since Mos is stabilized by its Cdk1 phosphorylation at S3 [84]. Notably, when protein synthesis is blocked, Cyclin B injection induces meiosis resumption in the absence of progesterone. Conversely, the injection of Mos at its physiological concentration is unable to activate Cdk1 under these conditions, despite its ability to activate MAPK [5,12]. These results suggest that some unidentified Class I proteins must be translated to activate Cdk1. Importantly, Class I proteins should be translated temporally before and independently of Cdk1 activation, and the suppression of their translation is expected to delay meiosis resumption, i.e., the time of NEBD (Table 4).

### 4.2. The Regulation of Translation by cis-Acting Elements and Trans-Acting Factors

Another caveat relies on the fact that the molecular regulation of translation during oocyte meiotic maturation is not fully understood. Seminal works on the plasminogen activator tPA have shown that, during meiosis resumption, tPA mRNA undergoes polyadenylation and increases translation, under the control of the information encoded in the 3′-UTR [87]. Importantly, blocking the process of poly(A) tail elongation with cordycepin (3′-deoxyadenosine) suppresses progesterone-induced meiotic maturation [88], suggesting that polyadenylation is necessary for translation of mRNAs that are required to resume meiosis. Many studies based on candidate approaches have focused on how interactions between *cis*-acting elements in the mRNA sequence and trans-acting factors, mainly RNA-binding proteins (RBPs), control polyadenylation.

*Cis*-acting elements in the 3′-UTR include cytoplasmic polyadenylation elements (CPEs) [89,90], polyadenylation responsive elements (PREs) [86], and Pumilio-binding element (PBE) [91]. The presence of CPEs has been proposed to be required and sufficient to drive translation in oocytes [92]. This regulation depends on the position and the number of CPEs [92]. While two CPEs located less than 50 nt apart are able to inhibit translation in prophase-arrested oocytes, translation activation during meiosis resumption likely depends on the presence of one CPE within 100 nt from the PAS [92]. Additionally, PBE contributes to the inhibition of translation of some mRNAs in prophase-arrested oocytes, including Cyclin B1 mRNA [91,92]. Since these results have been obtained by studying short 3′-UTR fragments derived from a few transcripts, an important challenge is to evaluate whether these rules can be extended to the general translation control. Other results suggest that a PRE, and not CPEs, is necessary and sufficient to promote the early translation of Mos in response to progesterone [86]. More recently, an attempt of reconciliation of these findings has proposed that the PRE modulates the use of different CPEs in Mos 3′-UTR by modifying the 3D-structures of the mRNA [93]. However, it is still unclear whether this mechanism is specific to Mos 3′-UTR and whether other *cis*-acting elements are involved in the regulation of the “early” wave of translation.

In the last two decades, many studies have focused on understanding how translation is controlled by *trans*-acting factors, in particular, RBPs. However, the inability to identify univocally the *cis*-acting elements regulating translation has resulted in the impossibility to ascertain whether CPE-binding protein 1 (CPEB1) [94], PRE-binding protein Musashi [95], or any other unknown RBPs that bind unidentified *cis*-acting elements are involved in the control of translation. Additionally, how the activities of the well-studied CPEB1 and Musashi are regulated remains controversial. At first, it was proposed that progesterone activates the kinase Aurora A, which, in turn, phosphorylates CPEB1 at S174 in order to allow Mos mRNA polyadenylation [96]. These results have been challenged as Aurora-A is unlikely activated before Cdk1 activation and seems dispensable for both CPEB1 phosphorylation and NEBD [97,98,99,100]. Moreover, in other vertebrates (mouse and porcine), Aurora-A does not control CPEB1 phosphorylation [101,102]. A second hypothesis is that Speedy/Ringo, whose translation is regulated by the Pumilio/Dazl complex, binds to Cdk1 and activates either CPEB1 [103] or Musashi [95,104]. Hence, a clear definition of the RBP network regulating the early wave of translation is still missing.

### 4.3. A Genome-Wide Overview of the Early Translation Wave: A Challenge for the Future

Noteworthy, genome-wide analysis has failed to identify proteins that are translated in response to progesterone since little changes in poly(A) tail length, polysome fractions, or in the proteome are observed in oocytes analyzed 90 min after the hormonal stimulation [70,72] (Supplementary Materials, Figure S1). Additionally, no increase in the poly(A) tail length and the protein expression level of either Mos, Cyclin B, or Speedy/Ringo, nor CPEB1 phosphorylation at S174 during meiosis resumption, has been recorded in the genome-wide analysis performed until now [70,72]. A possible explanation is that translation is not yet activated at 90 min after progesterone stimulation. As described above, a clear vision of how PKA downregulation regulates protein synthesis to activate Cdk1 activation during meiosis is still missing. The identification of the molecular mechanisms regulating translation in *cis* and in *trans,* as well as the identity of the synthesized proteins involved in Cdk1 activation, is one of the major open challenges in the field.

## 5. Progression and Completion of Meiotic Maturation: Shedding Light on the Cytoplasmic Maturation

Late meiotic maturation spans from NEBD to the arrest in metaphase II. In all species, activation of the late wave of translation is required for the MI to MII transition and to accumulate proteins required after fertilization.

### 5.1. Genome-Wide Description of the Late Steps of Meiotic Maturation

**Figure 4:**
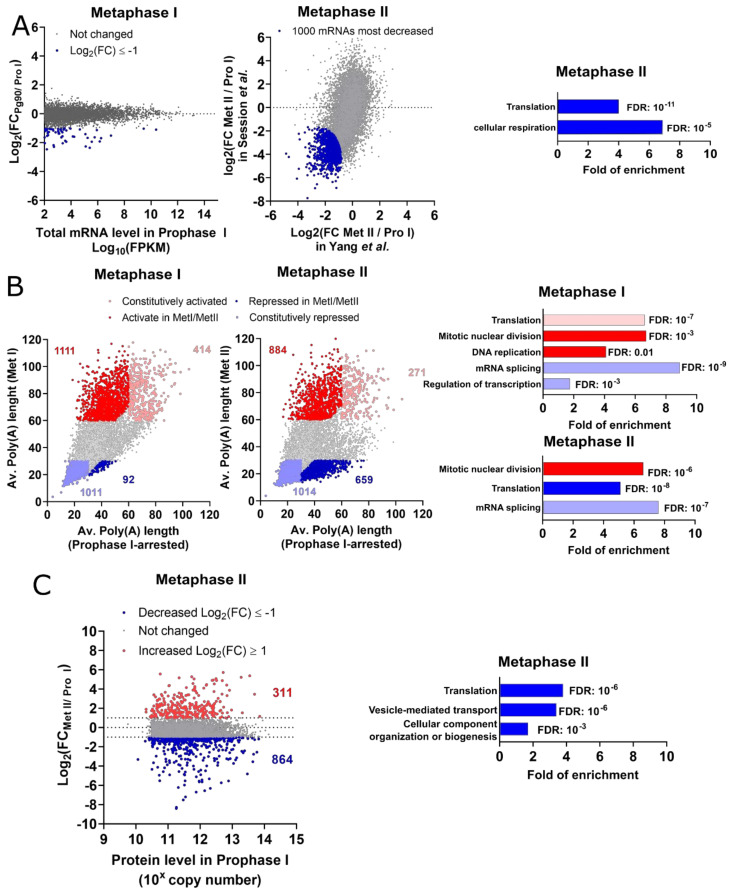
Genome-wide analysis of oocytes during meiotic maturation. (**A**) Changes in total mRNA levels [37,72]. Left panel: Changes between prophase I and metaphase I oocytes. Blue: mRNAs, whose level decreases more than 2-fold. Middle panel: Changes between prophase I (Pro I) and metaphase II (Met II) oocytes. The average rank is used to highlight the 1000 transcripts whose levels decrease the most during this period of meiotic maturation (blue). Right panel: Selection of the gene ontology terms enriched among the 1000 mRNAs from the middle panel. (**B**) Changes in the poly(A) tail length of each mRNA during meiotic maturation [72]. A color-code depicts 4 groups of transcripts. Light red: constitutively activated transcripts with poly(A) tail ≥ 60 nt at all periods; dark red: activated mRNAs whose poly(A) tail increases, at least, by 10 nt and is longer than 60 nt in Met I/Met II; light blue: constitutively repressed mRNAs with poly(A) tail ≤ 30 nt at all periods; dark blue: repressed mRNAs whose poly(A) tail decreases more than 10 nt and is shorter than 30 nt in Met I/Met II. Left panel: Changes between prophase I and metaphase I (Met I) oocytes. Middle panel: Changes between prophase I and metaphase II (Met II) oocytes. Right panel: Selection of the gene ontology terms enriched among the 4 groups of transcripts. (**C**) Left panel: Changes in the protein levels between prophase I and metaphase II oocytes [70]. The proteins whose copy number increases or decreases more than 2-fold are colored in red or blue, respectively. Right panel: Selection of the gene ontology terms enriched among the proteins whose level decreases more than 2-fold, from the right panel.

The full activation of Cdk1 induces NEBD and massive activation of protein translation (late wave of translation) that was first described by the incorporation of radiolabeled leucine [105,106]. De novo protein synthesis is required for meiosis progression, as preventing protein synthesis with cycloheximide at the time of NEBD abolishes entry into meiosis II [13,24,77]. Under this condition, oocytes enter a pseudo-interphasic state and replicate their genome [13,24]. While mRNAs are globally stable during meiosis I (Figure 4A), the activation of translation is accompanied by the extension of poly(A) tail of 1111 transcripts. The translation of 414 other transcripts remains high since prophase I (Figure 4B). At NEBD time, the translation wave produces components, regulating M-phase progression and DNA replication (Figure 4B). The analysis of the translation efficiency has provided similar results (Supplementary Materials, Figure S2). Among the M-phase regulators whose translation is upregulated are Aurora-A, Kif11/Eg5, and Wee1. These findings are in good agreement with the known functions and regulations of these proteins. Aurora-A accumulates in a Cdk1-dependent manner at NEBD time [97]. Kif11/Eg5 mRNA becomes highly polyadenylated, and the protein plays a critical role during the MI-MII transition [99]. Wee1 accumulates after NEBD and exerts its functions during the embryonic cell cycles [66]. Among the components of the DNA replication machinery is Cdc6, which is the only missing component of the pre-replication complexes in prophase-arrested oocytes [22,23]. Cdc6 accumulation after NEBD is necessary to support DNA replication during the embryonic cell cycles [22,23,24]. Importantly, the translation of Cdc6 and Wee1 must be tightly controlled as their precocious expression prevents the proper progression in meiosis [24,66].

While oocytes progress in meiosis II, an extensive wave of mRNA de-adenylation and degradation is also taking place (Figure 4A,B). The de-adenylated and degraded transcripts, commonly identified in Session et al. [37] and Yang et al. [72] datasets, are components of the translation machinery, such as mRNAs encoding the eukaryotic initiation factor (eIF) family and ribosomal proteins [107], as well as proteins involved in cellular respiration (Supplementary Materials, Figure S2). This result is also confirmed at the protein level as proteins involved in translation and vesicle-mediated transport components disappear from the proteome (Figure 4C). The decrease in the translation of ribosomal proteins during late meiosis resumption is also conserved in the mouse model [108]. Conversely, transcripts involved in mitotic nuclear division remain highly polyadenylated. With these new signatures, the fertilizable oocyte terminates the gene expression program that was initiated during early oogenesis.

### 5.2. The Role of Polyadenylation in the Control of the Late Wave of Translation

**Figure 5:**
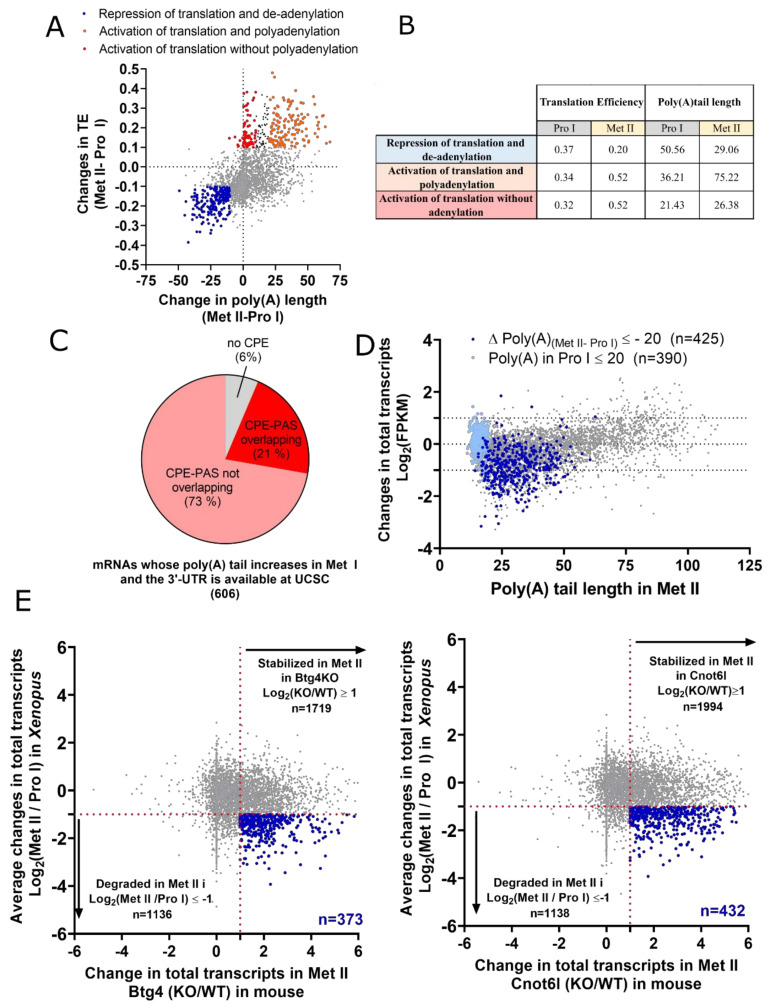
The relationship between poly(A) tail length, translation, and mRNA degradation. (**A**) Changes in translation efficiency (TE) and in the poly(A) tail length between fully-grown prophase (Pro I) and metaphase II (Met II) oocytes [72]. Transcripts are divided into three groups: transcripts whose poly(A) tail decreases, at least, by 10 nt and TE decreases, at least, by 0.1 (blue—repression of translation and de-adenylation), transcripts whose poly(A) tail increases less than 10 nt and TE increases more than 0.1 (red—activation of translation without polyadenylation), and mRNAs whose poly(A) tail increases more than 20 nt and TE increases, at least, by 0.1 (orange—activation of translation and polyadenylation). (**B**) Median values of translation efficiency and poly(A) tail length of the three groups described in panel A. (**C**) 3′-UTRs of the mRNAs whose poly(A) tail increases, at least, by 10 nt and is longer than 60 nt in metaphase I (Met I) oocytes are retrieved from the UCSC database. Sequences of cytoplasmic polyadenylation elements (CPEs) and CPE overlapping the PAS from Pique et al. [92] are searched within the 3′-UTRs. The pie chart represents the percentage of transcripts containing at least one CPE or one CPE overlapping the PAS or none. (**D**) Changes in total mRNA levels between fully-grown prophase (Pro I) and metaphase II (Met II) oocytes are compared to the length of the poly(A) tail in Met II oocytes [72]. A color-code depicts two groups. Light blue: mRNAs with a poly(A) tail length ≤ 20 in both Pro I and Met II oocytes; dark blue: mRNAs whose poly(A) tail length decreases, at least, by 20 nt between both stages. (**E**) Changes in the total mRNA levels between fully-grown prophase I (Pro I) and metaphase II (Met II) in *Xenopus* oocytes [72] are compared with mRNAs that are stabilized in btg4 (left panel) or cnot6l (right panel) knockout mouse Met II oocytes [114]. Blue: transcripts degraded in *Xenopus* Met II oocytes and stabilized in btg4 (left panel) or cnot6l (right panel) knock-out mouse Met II oocytes.

Extension of the poly(A) tail is widely believed to be the major mechanism, regulating the activation of translation during meiosis, as it has been demonstrated for Cyclin B1, Cyclin B4, Mos, Cyclin A, Aurora, and others [85,86,109]. A still open question in the field regards the absolute requirement of polyadenylation to activate translation. Genome-wide correlation between datasets published by Yang et al. on polyadenylated mRNAs and translation efficiency shows that de-adenylation is always correlated with the repression of translation [72] (Figure 5A,B). Furthermore, two groups of transcripts, whose translation activation occurs with the same efficiency, can be identified. The first one (142 transcripts) displays a strong increase of the poly(A) tail, while the second one does not show polyadenylation (70 transcripts), suggesting that translation activation of some transcripts occurs independently of polyadenylation (Figure 5A,B), as previously reported using candidate-approaches [110]. In addition, polyadenylation is not only unnecessary for translation activation but is also unlikely to be sufficient, as some transcripts are polyadenylated without activation of their translation (Figure 5A). This correlation analysis is, however, limited by the relatively small number of transcripts, whose polyadenylation status and translation efficiency are both known during meiosis. The validation of transcripts whose activation of translation is correlated or not with polyadenylation will be a mandatory step to understand the molecular mechanisms underlying the activation of translation that occurs independently of poly(A) tail elongation during meiosis.

### 5.3. The RBP Network Controlling the Late Wave of Translation

It has been proposed that CPEB1 degradation controls the activation of the “late” wave of translation that occurs at NEBD. CPEB1 degradation by the Skp, Cullin, F-box containing (SCF) ubiquitin ligase pathway depends on CPEB1 phosphorylation by Cdk1 in *Xenopus* [82,111,112]. This phosphorylation event is conserved in the mouse as both Cdk1 and MAPK cooperate to phosphorylate CPEB1 [102,113,114]. The injection of a CPEB1 mutant, which can be neither phosphorylated nor degraded (6A-CPEB1), does not impact meiosis resumption but strongly abolishes the MI-MII transition, demonstrating that these CPEB1 modifications are critical for meiosis progression [82]. Likewise, these data suggest that CPEB1 inhibits the translation of some Class II mRNAs that are required for meiosis progression. At NEBD, the repression previously exerted by CPEB1 is relieved upon its degradation, hence resulting in the activation of translation. While it is well established that CPEB1 plays a central role in controlling translation in both *Xenopus* and mouse oocytes, discrepancies still exist regarding the identity and the UTRs features of mRNAs that are translated following CPEB1 degradation. It was proposed that the binding of CPEB1 to a CPE overlapping the PAS could prevent CPSF-dependent polyadenylation until CPEB1 gets degraded at NEBD [92]. However, the poly(A) database from Yang et al. [72] shows that only 21% (129/606) of the transcripts, whose poly(A) tail length increases in metaphase I and whose UTR is deposited at UCSC database, contains a CPE overlapping the PAS in the 3′-UTR (Figure 5C). Therefore, additional mechanisms should explain the temporal activation of polyadenylation of late transcripts. In *Xenopus*, the Cyclin B1 mRNA, an essential transcript required for meiosis resumption, would be translated in a CPEB1 degradation-dependent manner [82,92]. However, Cyclin B1 accumulates in response to progesterone before and independently of Cdk1 activation [16]; its translation is, therefore, likely independent of CPEB1 degradation. Further studies using unbiased approaches will be required to globally identify mRNAs belonging to the late wave of translation. This knowledge will be the first step to functionally determine the sub-group of late translated transcripts that are important for meiosis progression.

Besides CPE and PBE, other *cis*-acting elements have been involved in the control of the late wave of translation. The 3′-UTR of Wee1 mRNA contains a translational control sequence (TCS) that binds to Zar2 (Zygote-arrest 2) in prophase oocytes and represses its translation [25,115]. As for CPEB1, Zar2 is degraded at NEBD time, when Wee1 translation is initiated [25]. Zar2 and CPEB1 act in a similar manner—their degradation is necessary to remove the repression of translation operating in prophase. Using an approach based on 3′-UTR reporters, it has been shown that the removal of the inhibitory complex (de-repression) increases the rate of translation in prophase I-arrested oocytes [76]. Importantly, the translation of these reporters further increases during meiosis resumption, suggesting that the activation of the translation does not simply rely on the de-repression of mRNAs but requires the recruitment of activating complexes [76]. Interestingly, CPEB4 is a Class II protein that has been proposed to be part of the activating complex required for the late polyadenylation [18]. Accordingly, when CPEB4 translation is inhibited, oocytes cannot enter metaphase II but, instead, enter into pseudo-replicative interphase without extruding the first polar body [18].

Altogether, the code of *cis*-acting elements cooperates with differentially expressed RBPs. These RBPs undergo specific cell cycle-dependent modifications and tightly orchestrate the late wave of translation that includes proteins required for meiosis progression (Class II) and the development of the embryo (Class III).

### 5.4. De-Adenylation and RNA Degradation during Meiosis II

An extensive wave of mRNA de-adenylation and degradation takes place during the MI-MII transition (Figure 4A,B), as also shown in mouse [116,117]. In the last years, many studies using the mouse model have been conducted to elucidate the composition of the machinery involved in mRNA de-adenylation/degradation, the temporal control of its activation, and its specificity. It has been established that the CCR4-NOT (Carbon Catabolite Repression—Negative On TATA-less) de-adenylation complexes, composed of Btg4-Cnot7/8 and ZFP36L2-Cnot6l [114,118], as well as the de-capping proteins DCP1A and DCP2, play a central role in these mechanisms [119]. Interestingly, Cdk1 not only promotes the translation of the components of the de-adenylation machinery, it further modulates the activity of these proteins by phosphorylation, thus enabling a precise coupling between mRNA de-adenylation/degradation and meiosis progression. The translation of Btg4, Cnot8, Cnot6L, DCP1A, and DCP2 is activated during meiosis, demonstrating that stockpiled mRNA degradation is encoded by the translation program of the oocyte [76,113,118]. Additionally, Cdk1 phosphorylates Y-box binding protein 2 (YBX2) [120,121], as well as DCP1A and DCP2 [119], which, respectively, results in mRNA deprotection and activates the de-capping activity of DCP1A and DCP2. Another layer of RNA regulation involves adenosine methylation that converts adenosine into *N*^6^-methyladenosine (m^6^A). Indeed, it was proposed that YTHDF2 binds m^6^A and precisely selects mRNAs to be degraded [122]. In contrast with these protein-based mechanisms, microRNA-mediated RNA degradation is predominant at the time of zygote genome activation after fertilization [123].

In *Xenopus* oocytes, 80% of the mRNAs whose level decreases more than 2 folds have poly(A) tail shorter than 40 nt (Figure 5D). Hence, the length of poly(A) tail could also play a role in mRNAs degradation, although a large group of mRNAs with short poly(A) are stable (Figure 5D). This apparent discrepancy is explained by the presence of two groups of mRNAs with short poly(A) tails in metaphase II: mRNAs whose translation is already repressed since prophase (Figure 5D, light blue) and mRNAs that undergo de-adenylation during meiosis progression (Figure 5D, dark blue). mRNAs of the first group, which are stable during meiosis, are probably stockpiled to support embryo development, while mRNAs of the second group are degraded. Therefore, the dynamics of de-adenylation is a more important predictor of mRNA degradation than the length of the poly(A) tail itself. In *Xenopus* oocytes, the molecular composition of the de-adenylation complex is still unknown, but it could involve the zinc finger protein 36 C3H-like type 2 (zfp36l2), whose synthesis increases during meiosis [28]. Moreover, both Btg4 and Cnot6l mRNAs are polyadenylated [72], opening the interesting possibility that their function is conserved among vertebrates. Accordingly, around 30% of mRNAs that are degraded in *Xenopus* oocytes at metaphase II have homologous mRNAs in mice that are stabilized either in the Btg4^−/−^ or Cnot6l^−/−^ metaphase II oocytes [114] (Figure 5E). This observation strengthens the idea that both Btg4 and Cnot6l control the degradation of stockpiled mRNAs in both *Xenopus* and mouse. Only a few studies have addressed how mRNAs are selected to be degraded in *Xenopus* oocytes. It was proposed that mRNA de-adenylation occurs as a default mechanism for mRNAs that do not contain CPE elements [124], as described in mouse [76]. Other works instead suggest that de-adenylation is activated by the presence of (A + U)-rich elements (ARE) in the 3′UTR of mRNAs [28].

## 6. Conclusions and Perspectives

**Figure 6:**
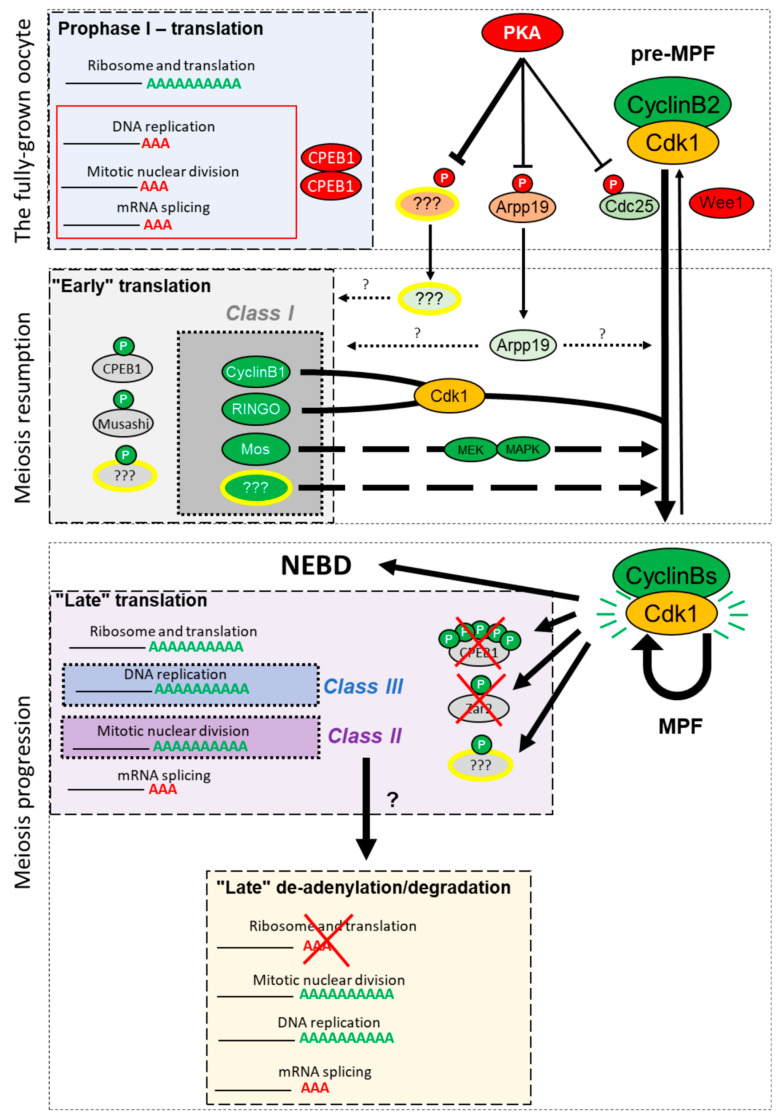
The bidirectional regulation between protein translation and meiosis progression. The fully-grown prophase-arrested oocyte actively translates proteins related to cell metabolism and ribosome biogenesis. mRNAs encoding proteins involved in M-phase progression, DNA replication, and mRNA splicing exhibit short poly(A) tails and are stored for future translation. The prophase arrest is maintained by the high activity of protein kinase A (PKA) and the phosphorylation of its substrates, which indirectly inhibit the activation of the M-phase promoting factor (MPF). In the vertebrates, the prophase arrest is released by a decrease of PKA activity and the dephosphorylation of PKA substrates. De novo protein synthesis occurs before NEBD (“Early” translation), and a sub-group of the de novo synthesized proteins is required to activate Cdk1 (Class I). Two RNA-binding proteins (RBPs) have been involved in the activation of the early translation wave—CPEB and Musashi. Once activated, Cdk1 activates the “late translation” (purple box). This second translation wave involves the phosphorylation and degradation of inhibitory RBPs, including CPEB1 and Zar2. The translated proteins are involved in meiosis progression (Class II), support embryonic cell divisions, such as components of the DNA replication machinery (Class III), and further contribute to regulating RNA translation and degradation by de-adenylation in metaphase II. Among the transcripts that are de-adenylated are ribosome compounds and translation initiation factors.

The understanding of the regulation of the G_2_/M transition has greatly benefited from the comparative studies using various model organisms, such as *Xenopus*, mouse, *Drosophila*, and starfish. Nevertheless, many questions are still open regarding the molecular switch that leads to Cdk1 activation (Figure 6). One of the major challenges is to solve the “black box” of meiosis, discovering what are the mechanisms controlling mRNA translation, what is the identity of the translated proteins, what are their functions in orchestrating meiosis. By combining the emerging inputs of omics techniques with the power of biochemistry, the *Xenopus* oocytes system opens a new era of investigations, in which genome-wide analysis is coupled to the detailed molecular investigation.

Each genome-wide dataset describes meiotic maturation from slightly different perspectives, with specific strengths and weaknesses. The transcriptome analysis is extremely sensitive, but it does not distinguish between mRNAs that are used immediately or stockpiled for further use. The poly(A) dataset provides a snapshot of the active translation landscape at a precise moment, but it fails at classifying mRNAs with a very low level of expression. Moreover, it is based on the assumption that poly(A) tail length is the dominant mechanism regulating translation. The proteome records the steady-state between translation regulation and protein turnover, but its sensitivity is lower than the other two approaches, as it fails to sense many regulators of cell division that are under the threshold of detection. Clearly, the integration of all the information derived from transcriptome, poly(A) dataset, translatome, and proteome is required to fully understand the physiological dynamics of translation during each phase of oogenesis. This will set the ground to investigate the molecular mechanisms driving the transitions the oocyte undergoes in its journey to becoming a fertilizable egg.

Recent works have revealed that the correlation between the levels of mRNAs and their encoded proteins is far from being optimal in many biological systems [125,126,127]. Therefore, the post-transcriptional mechanisms regulating gene expression identified during meiosis should play fundamental functions in sculpting the proteome in many systems beyond oocytes.

### Methods—Analysis of Published Datasets

Raw data from transcriptome data during oogenesis used in Figure 2 was from Session et al. [37]. The oocyte transcriptome raw datasets for fully-grown prophase-arrested oocytes and metaphase II arrested oocytes used in Figure 3 and Figure 4 were from Session et al. [37] and Yang et al. [72]. TPM stands for a transcript per million, and FPKM for fragment per kilobase per million. The datasets of poly(A) length used in Figure 3B,C and Figure 4B and the translation efficiency used in Figure 3B were obtained from Yang et al. [72]. The protein counts in fully-grown prophase-arrested oocytes and in metaphase II arrested oocytes used in Figure 3C and Figure 4C were obtained from the proteomic dataset published by Peuchen et al. [70]. All the data analysis has been performed in Excel, and graphs have been prepared using Prism 8. The gene-ontology analysis in Figure 2B, Figure 3B,D,F and Figure 4A–C was performed with the online version of Panther using the “GO biological process complete” as an annotation dataset, as the Fisher’s exact as a test type. The fold of enrichment and false discovery rate (FDR) were plotted using Prism 8. The comparison between the mRNAs that are degraded in *Xenopus* metaphase II oocytes and the mRNAs stabilized in btg4^−/−^ or cnot6l^−/−^ mouse metaphase II oocytes was performed in Excel. The raw dataset was obtained from Sha et al. [114].

## Figures and Tables

**Table 1:** Temporal and functional classification of proteins during meiosis. Summary of the known proteins that accumulate during meiosis before (early) or after (late) NEDB. Class I proteins are involved in Cdk1 activation. Class II proteins are important for meiosis progression, and Class III proteins support embryo development.

Protein	Time of Translation	Classification
**Speedy/RINGO**	?	Class I [8,9]
**Mos**	Early	Class I [10,11,12,13,14,15]
**Cyclin B1**		Class I [5,16]
**Xkid**		Class II [17]
**CPEB4**	Late	Class II [18]
**Bub1**		Class II [19]
**Erp1/Emi2**		Class II [20]
**TPX2**	?	Class II [21]
**Cdc6**	Late	Class III [22,23,24]
**Wee1**		Class III [25]
**Cdt1**		Class III [26]
**Cyclin E1**		Class III [27]
**Zfp36l2**		? [28]
**Drosha**	?	? [29]
**Lamin L1**	?	Class III [30]

Xkid: Xenopus kinesin-like DNA binding protein, CPEB4: CPE-binding protein 4, TPX2: targeting protein for Xklp2, and Zfp36l2: Zinc Finger Protein 36 C3H1 Type-Like 2.

**Table 2:** PKA-substrates in *Xenopus* prophase-arrested oocytes (Prophase I). Summary of the features of the PKA substrates identified so far. OA: Okadaic Acid (pharmacological inhibitor of PP2A-B55δ, the phosphatase counteracting active MPF). MPF injection refers to the injection of cytoplasm from metaphase II-arrested oocytes [1].

			Ability to Block Meiotic Maturation When Phosphorylated by PKA	
Protein	Expressed in *Xenopus* Prophase I	Time of Dephosphorylation	Progesterone or PKI	MPF Transfer or OA Injection
*Inhibitor 1 (I1)*	Unknown	Unknown	YES [60]	NO [60]
*Wee1*	NO [66]		Unknown	Unknown
*Cdc25C*	YES [69]	Late [63]		
*MpP-32*	YES [47]	Late [47]		
*Arpp19/MpP-20*	YES [47,49]	Early [47,49]	YES [49]	NO [49]

**Table 3:** Putative PKA substrates in fully-grown *Xenopus* oocytes. Proteins bearing a PKA consensus site (R/K-R/K-X-S/T-X_hydrophobic_) in which the phosphorylation level of the S/T residue at position 4 of the motif decreases in the response of progesterone (Pg). “Phospho (STY) probabilities” estimate the likelihood of the residue highlighted in red to be phosphorylated. “FC (Pg90/ProI)” is the fold change of the level of phosphorylation detected in oocytes stimulated for 90 min with progesterone as compared to unstimulated prophase-arrested oocytes. In green are highlighted the genes whose function is connected to the control of protein translation.

Gene Names	Proteins	Predicted PKA Site	(STY) Probabilities	Fold Change (Pg90/ProI)	Protein Functions
*akt1s1*	*Proline rich substrate of Akt*	DETSKFPS(237)PDLDRIA	0.955	0.353	Subunit of mTORC1, a master regulator of protein synthesis
*clspn*	*Claspin*	ADNVKGHS(88)DNEENEE	1	0.131	Cell cycle checkpoints
*gly*	*Glycogen* *synthase*	HRRSKKGS(700)IDATNSS	0.271	0.401	Metabolism of glycogen
*hadha*	*Hydroxyacyl-CoA dehydrogenase*	NDKVKKKS(413)VTSFERD	0.751	0.476	Fatty acids metabolism
*rif1*	*Replication timing regulatory factor 1*	SWRSKTKS(1484)IEKDDNV	0.994	0.336	Telomere-associated protein
*rps6*	*Ribosomal protein S6*	IAKRRRLSS(236)LRAS(240)TSKSESS	0.455/0.545/0.989	0.344	Cell growth and proliferation through mRNA translation
*serpinA2*	*Serpin* *A2*	FFNKKKLS(133)ELQVHEA	0.986	0.301	Inhibition of serine proteases
*spats2*	*Spermatogenesis associated, serine rich 2*	NNKTTRSGS(218)LSSSSQSL	0.461	0.227	RNA binding protein involved in male meiosis
*ube2o*	*Ubiquitin ligase*	SGTGRKKS(496)IPLSIRN	1	0.393	Ubiquitin-protein ligase

**Table 4:** Class I proteins whose translation is required for MPF activation in *Xenopus* oocytes. Summary of the expected features of the Class I proteins and those of the Class I proteins already identified. CHX: cycloheximide.

	Timing		Protein Accumulation	Effects on NEBD		
	Polyadenylation	Protein Accumulation	Cdk1 Activity	Loss of Function	Gain of Function	Gain of Function + CHX
Class I	Early	Before NEBD	Not required	Delay/Block	Induction	Induction
*Cyclin B1*	Early [85]	Before NEBD [16]	Not required [16]	Delay [5]	Induction [5]	Induction [5]
*Mos*	Early [86]	At NEBD [16]	Required [16]	Delay [5,14,15]	Induction [11]	No Induction [5]
*Speedy/Ringo*	Unknown	Unknown	Unknown	Delay [67]	Induction [8,9]	Induction [8,9]

## Supplementary Materials

The following are available online at https://www.mdpi.com/2073-4409/9/6/1502/s1, Figure S1: Genome-wide analysis of fully-grown oocytes stimulated or not by progesterone for 90 minutes, Figure S2: Changes in translation efficiency during meiosis resumption and progression.
