# Supplementary material for: Translational Control of Xenopus Oocyte Meiosis: Toward the Genomic Era
Source: Cells. 2020 Jun 19;9(6):1502. doi: 10.3390/cells9061502 (PMC7348711; doi:10.3390/cells9061502)
Supplement: Supplementary file 1 [file cells-09-01502-s001.pdf]

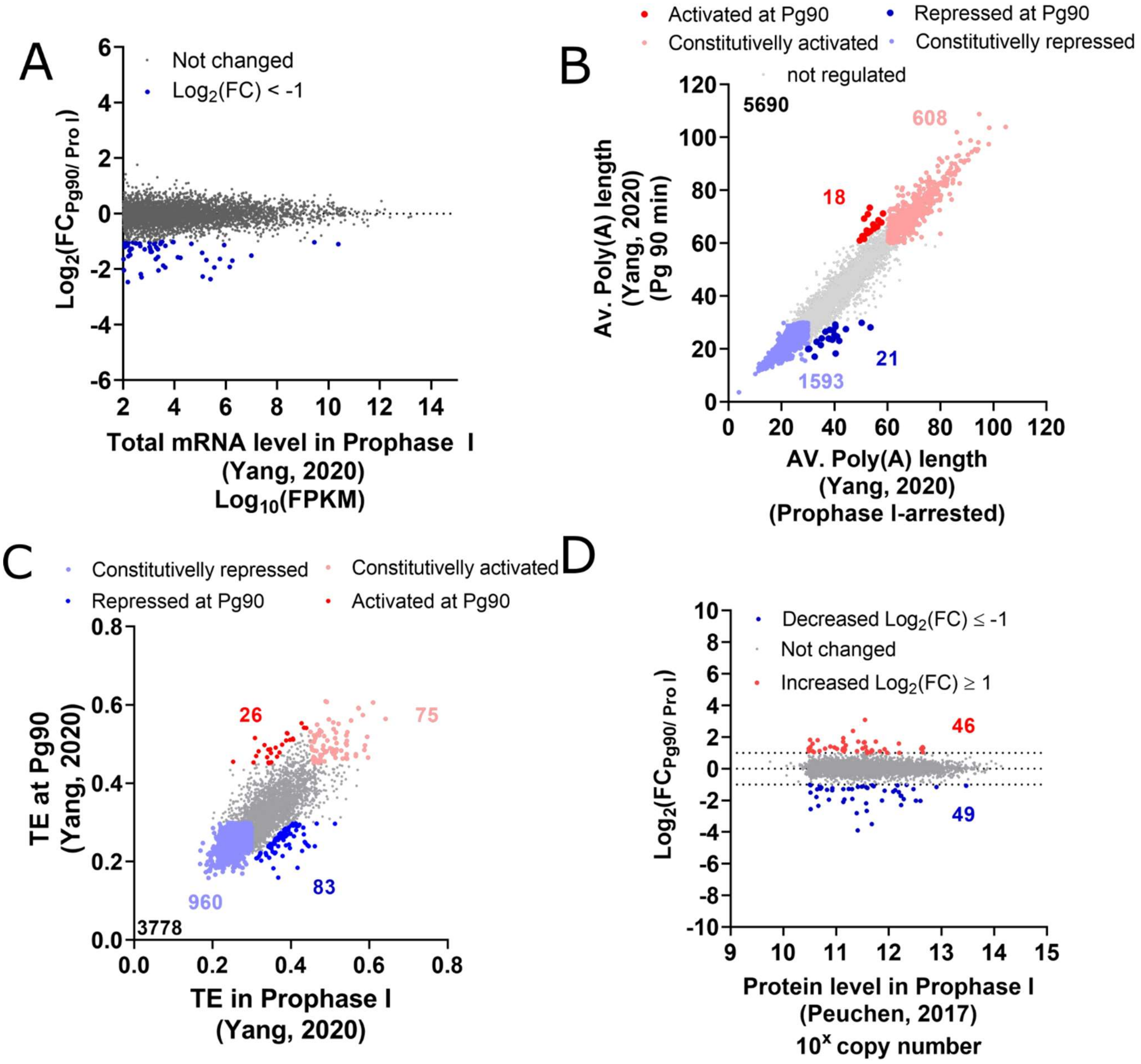

Supplementary Figure 1. Genome-wide analysis of fully-grown oocytes stimulated or not by progesterone for 90 minutes. Fully-grown oocytes are referred as "Pro I". **A)** Changes in total mRNA levels quantified using the dataset published by Yang *et. al.*. Blue: mRNAs whose level decreases more than 2-fold. **B)** Changes in the poly(A) tail length of each mRNA. Light red: constitutively activated transcripts with poly(A) tail ≥ 60 nt at both oocyte stages; dark red: activated mRNAs whose poly(A) tail increases of at least 10 nt and is longer than 60 nt in progesterone-treated oocytes; light blue: constitutively repressed mRNAs with poly(A) tail ≤ 30 nt at both oocyte stages; dark blue: repressed mRNAs whose poly(A) tail decreases more than 10 nt and is shorter than 30 nt in progesterone-treated oocytes. **C)** Translation efficiency (TE) of each mRNA. A color-code depicts 4 groups. Light red: the constitutively activated transcripts with TE ≥ 0.45 at both oocyte stages; dark red: activated mRNAs whose TE increases more than 0.1 and is higher than 0.45 in progesterone-treated oocytes; light blue: constitutively repressed mRNAs with a TE ≤ 0.3 at both oocyte stages; dark blue: repressed mRNAs whose TE decreases more than 0.1 in progesterone-treated oocytes. Raw data were derived from Yang *et al.* . **D)** Changes in the protein levels were calculated using the dataset published by Peuchen *at al.* . The proteins whose copy number increases or decreases more than 2-fold were colored in red or in blue, respectively.

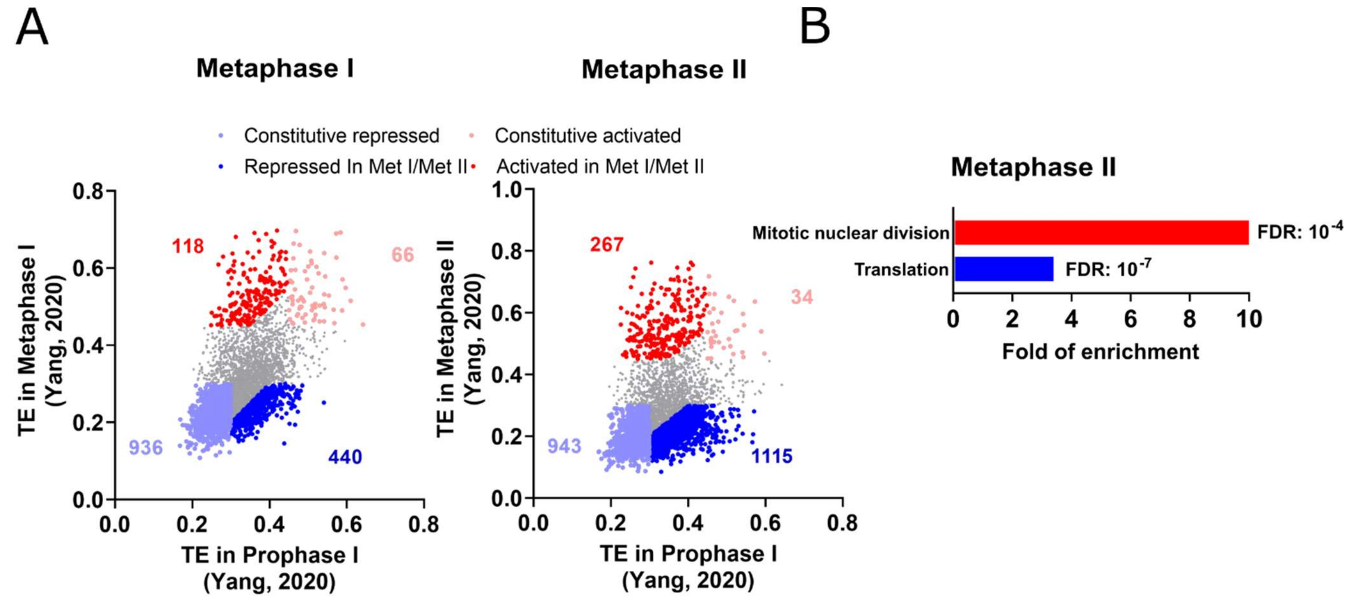

Supplementary Figure 2. Changes in translation efficiency during meiosis resumption and progression. Fully-grown prophase oocytes (Pro I) are compared with either metaphase I oocytes or metaphase II oocytes. **A)** Translation efficiency (TE) of each mRNA. A color-code depicts 4 groups. Light red: the constitutive activated transcripts with TE ≥ 0.45 in both Pro I and metaphase I or II stages; dark red: activated mRNAs whose TE increases more than 0.1 and is higher than 0.45 in metaphase I or metaphase II oocytes; light blue: constitutively repressed mRNAs with a TE ≤ 0.3 in both Pro I and metaphase I or II stages; dark blue: repressed mRNAs whose TE decreases more than 0.1 in metaphase I and metaphase II oocytes. Quantification using the dataset published by Yang *el al.* . **B)** Selection of the gene ontology terms enriched among the 2 groups of transcripts identified in panel A, displayed using the same color-code.
